# Pain, Substance Use Disorders, Mental Health, and Buprenorphine Treatment among Patients With and Without HIV

**DOI:** 10.1007/s10461-024-04494-w

**Published:** 2024-09-12

**Authors:** Emily A. Miller, Kathleen A. McGinnis, E. Jennifer Edelman, Termeh Feinberg, Kirsha S. Gordon, Robert D. Kerns, Brandon D. L. Marshall, Julie A. Patterson, MaryPeace McRae

**Affiliations:** 1 Department of Pharmacotherapy and Outcomes Science, Virginia Commonwealth University School of Pharmacy, Richmond, VA USA; 2 VA Connecticut Healthcare System, West Haven, CT USA; 3 Section of General Internal Medicine, Yale University School of Medicine, New Haven, CT USA; 4 Kelly Government Solutions, Silver Spring, MD USA; 5 Department of Biostatistics, Yale University School of Public Health, New Haven, CT USA; 6 Center for Medical Informatics, Yale University School of Medicine, New Haven, CT USA; 7 Department of Psychiatry, Yale University School of Medicine, 300 George Street, Suite 901, New Haven, CT USA; 8 Department of Epidemiology, Brown University School of Public Health, Providence, RI USA; 9 410 North 12th Street, Richmond, VA 23298 USA

**Keywords:** HIV, Opioid use disorder, Population analysis, Buprenorphine, Veterans, Mental health

## Abstract

Treatment of opioid use disorder (OUD) with buprenorphine improves outcomes and mortality among people with HIV (PWH). However, engagement is low and is influenced by comorbidities. We examined the impact of patterns of co-occurring pain, substance use disorders (SUDs), and mental health diagnoses on buprenorphine initiation and retention in PWH. The Veterans Aging Cohort Study contained 7,875 patients (2,702 PWH and 5,173 without HIV) with new OUD clinical encounters (2008–2017). Buprenorphine initiation and retention were derived from prescription data. We identified patterns of co-occurring diagnoses (via ICD codes) and assessed the effects of class membership on both outcomes using latent class analysis and regression analyses. The mean age of patients was 55, 98% were male, 58% Black, 8% Hispanic, and only 8% initiated buprenorphine within 12 months of OUD diagnosis. Four classes of co-occurring diagnoses were identified: “Few Co-occurring Diagnoses” (42.3%); “Multiple Pain Conditions” (21.3%); “Pain + SUD” (18.4%) and “Pain + SUD + Mental Health” (18.0%). Patients in the “Pain + SUD” class and “Pain + SUD + Mental Health” class were significantly less likely to initiate buprenorphine and had 59% and 45% lower odds, respectively, of initiating buprenorphine compared with patients in the “Few Co-occurring Diagnoses” class; this effect did not vary by HIV status. Buprenorphine retention was not significantly associated with HIV status or class membership. However, Black Veterans were less likely to initiate or be retained in buprenorphine treatment. Higher comorbidity burden was negatively associated with buprenorphine initiation but not with retention. More research is warranted to determine other factors that may influence treatment retention.

## Introduction

The rate of opioid use disorder (OUD) is 2–4 times higher among persons with human immunodeficiency virus (PWH) compared to the national average [1–4]. Specifically, nationally representative estimates suggest that as many as 24,000–48,000 PWH in the United States meet the criteria for a current OUD diagnosis. Opioid agonist therapy includes buprenorphine and methadone. Both medications are effective at reducing opioid cravings, opioid use, related overdose and mortality [5]. Additionally, treatment of OUD improves HIV-related outcomes, such as antiretroviral receipt and adherence as well as HIV viral suppression [6]. However, despite the effectiveness of opioid agonist therapy, engagement with opioid agonist therapy remains low among both PWH and persons without HIV (PWoH) [7, 8].

Numerous studies have described predictors of opioid agonist therapy initiation and retention among PWH and PWoH. Previous research suggests that some co-occurring mental health conditions, including bipolar disorder and depression, may be associated with reduced buprenorphine initiation and retention [9–11], while post-traumatic stress disorder and anxiety disorder may have no impact on treatment engagement [9, 12, 13]. When the impact of co-occurring mental health conditions on OUD treatment engagement have been analyzed collectively (i.e., “any mental health conditions”), researchers have reported mixed effects, including decreased, increased, and no change in treatment engagement when considering mental health status [14–16]. Conflicting literature on the impact of non-opioid substance use disorders (SUDs), such as alcohol, cocaine, and cannabis use, on engagement in treatment for OUD has reported mixed findings regarding the effect of these co-occurring SUDs on initiation and retention [7, 9, 13, 16]. Finally, the role of pain on OUD treatment engagement has not been fully explored. Theoretically, pain may increase opioid craving [17], which, in turn, may increase the likelihood of OUD treatment discontinuation [18]. However, the observed effect of co-occurring pain diagnoses on treatment engagement is variable, with the presence of co-occurring pain diagnoses reducing the likelihood of buprenorphine initiation [10, 11] but not retention in treatment [16].

The conflicting literature on the effects of co-occurring pain, non-opioid SUDs, and mental health diagnoses, on treatment retention may reflect the complexities of the comorbidities individually or in combination [8, 16, 19–23]. Previous studies regarding SUDs have provided insight into substance use and SUD treatment by considering patient characteristics collectively [24–27]. Latent class analyses (LCA), which identify subgroups based on patterns of patient characteristics, have been used to investigate how patterns of pain, co-occurring substance use, and mental health diagnoses impact HIV medication adherence [28], social functioning [29], and health status [30]. However, LCA has not been widely used to help explain how patterns of these co-occurring conditions might impact either OUD treatment engagement or retention [31].

The objective of this research was to holistically examine the impact of co-occurring pain, SUDs, and mental health diagnoses on buprenorphine treatment engagement among patients with OUD. Specifically, we aimed to: (1) identify patterns of co-occurring pain, SUD diagnoses, and mental health conditions, among patients with OUD with and without HIV; (2) assess whether patterns of co-occurring diagnoses were associated with OUD treatment initiation and engagement; and (3) examine whether the relationship between patterns of co-occurring diagnoses and treatment engagement varied by HIV status.

## Materials and Methods

### Data and Analytic Sample

Electronic medical record data were obtained from the Veterans Aging Cohort Study (VACS), an observational cohort study of PWH (*n* = 55,984) and 1:2 matched PWoH (*n* = 116,705) receiving care at the Veterans Health Administration (VHA) from October 1996 to September 2017 [32]. Ascertainment of HIV status and matching in the cohort has been described elsewhere [14]. Within VACS, we identified patients with one or more new OUD clinical encounters from January 2008 to September 2017. An OUD behavioral treatment encounter was defined as an inpatient or outpatient encounter with a primary or secondary OUD diagnosis (ICD-9-CM codes 304.0x, 305.5x and 304.7x; ICD-10 codes F11.1x and F11.2x) [14]. We considered encounters to be “new” treatment episodes if the encounter was preceded by a one year period without opioid agonist therapy or an ICD code for OUD [14, 33]. January 2008 was selected as the earliest encounter date because access to buprenorphine was broadly expanded within the VHA system at that time [14, 34, 35]. Although methadone and naltrexone are also utilized for OUD, by 2017 the majority of Veterans that were receiving medications for the treatment of OUD (as many as 72%) were treated with buprenorphine [36, 37].

### Measures and Patient Characteristics

We examined the initiation of buprenorphine within one year of the earliest OUD clinical encounter (i.e., index date) using VHA Pharmacy Benefits Management records for any formulations of buprenorphine or buprenorphine/naloxone indicated for OUD [14]. Prescriptions for the buprenorphine transdermal patch, indicated for pain management, were excluded [14, 38]. As in previous studies, six- and 12-month buprenorphine retention were defined as *≥* 80% proportion of days covered in the six months and year following buprenorphine initiation, respectively [14, 39, 40].

Patient demographic and health characteristics, including age, sex, race/ethnicity (categorized as non-Hispanic White, non-Hispanic Black, Hispanic, and other), HIV status, smoking status (categorized as never, current, and past), and pain intensity rating closest to the first (index) OUD encounter date were obtained from the VHA Corporate Data Warehouse. Smoking status is assessed and documented annually on all VHA patients [41]; it was included given reported disparities in smoking status among VACS patients with SUDs with and without HIV [8], and the association between smoking status and OUD behavioral treatment intensity among VACS patients [42]. Self-reported pain intensity ratings, measured on a scale of 0 (no pain) to 10 (worst pain imaginable), were dichotomized as no pain/mild pain (less than 4) and moderate/severe pain (4 or greater) [43–45]. Among PWH, receipt of antiretroviral medications during the year before and after the index OUD encounter date were utilized. Four pain (fibromyalgia; headache; limb/extremity, joint, or arthritic pain; and neuropathic pain) [46], six non-opioid substance use (alcohol, cocaine, cannabis, amphetamines, sedatives, and hallucinogens), and six mental health (major depressive disorder, other depression, post-traumatic stress disorder, anxiety disorder, bipolar disorder, and schizophrenia) co-occurring diagnoses were selected based on past literature [13, 38]. To identify patients with common pain conditions specifically, diagnoses were ascertained from ICD-9 and ICD-10 codes using the crosswalk developed by Mayhew et al. [46].

### Statistical Analysis

To identify patterns of co-occurring pain conditions, SUDs, and mental health diagnoses in OUD patients with and without HIV, a LCA was utilized. LCA, which has previously been used to better understand SUDs, is a statistical method for identifying meaningful segments, or classes, within a population [24–27]. The LCA included sixteen indicators: four types of pain diagnoses; six SUDs, and six mental health diagnoses, as described above. The optimal number of classes was selected based on Bayesian Information Criteria (BIC), with consideration given to average posterior probability (i.e., the likelihood of class membership for each observation; a 70% threshold is well established as acceptable) [47], interpretability and class size (we required the smallest class to have at least 5% prevalence) [27]. The LCA was performed for two to six class solutions, after which increasing numbers of classes provided no benefit to fit statistics. Patients were assigned to a class for which they had the highest estimated probability of membership. Differences in class membership based on patient characteristics, including demographic and health characteristics, were analyzed using chi-square and ANOVA for categorical and continuous variables, respectively.

Bivariate and multivariable logistic regression analyses were then used to analyze the association of class membership with three outcomes: buprenorphine initiation within 12 months of the index OUD encounter and six- and 12-month retention among those who initiated buprenorphine. In adjusted multivariable models, age, sex, race/ethnicity, smoking status, and HIV status were included as covariates, with an HIV status-class membership interaction term. Multicollinearity was assessed using variance inflation factor testing. None of the variables included in the LCA had missing data. One covariate in the regression model had low levels of missing data (smoking status, missing = 0.3%); complete case analysis was used in the regression analyses. Stata (15.1; College Station, TX) was used for the analysis, with two-sided test of *p* < 0.05 denoting statistical significance.

## Results

### Sample Characteristics

During the baseline period of January 2008 to September 2017, a total of 7,875 patients had an index OUD clinical encounter, including 2,702 (34.3%) PWH and 5,173 PWoH (Table 1). The sample predominately consisted of non-Hispanic Black (58.2%), males (97.8%), and those who reported current smoking (79.6%). The most common past-year, co-occurring SUD diagnoses were alcohol (47.4%), cocaine (32.7%), and cannabis (15.5%). Several co-occurring pain and mental health conditions were more common among PWoH than PWH, including limb, joint, or arthritic pain (51.9% vs. 38.7%, Χ^2^ = 123.88, *p* < 0.001); neuropathic pain (22.1% vs. 15.9%, Χ^2^ = 42.43, *p* < 0.001); post-traumatic stress disorder (28.9% vs. 21.2%, Χ^2^ = 55.05, *p* < 0.001); and schizophrenia (10.9% vs. 8.5%, Χ^2^ = 10.50, *p* = 0.001). The majority of PWH in the sample were receiving antiretrovirals before their OUD index visit (70.4%) and this proportion increased following OUD diagnosis (78.0%, Χ^2^ = 1,400, *p* < 0.001).

**Table 1:** Demographic and health characteristics of patients with at least one index OUD episode by HIV status

Baseline Characteristics	Total Sample (*n* = 7,875)	PWH (*n* = 2,702)	PWoH (*n* = 5,173)	Test Statistic	*p*-value
Age, mean years (SD)	54.7 (7.77)	54.9 (7.57)	54.6 (7.88)	1.66	0.198
Male Sex, n (%)	7,704 (97.8)	2,623 (97.1)	5,081 (98.2)	10.96	0.001
Race/ethnicity, n (%)				38.47	< 0.001
White	2,536 (32.3)	768 (28.4)	1,768 (34.2)		
Black	4,583 (58.2)	1,626 (60.2)	2,957 (57.2)		
Hispanic	657 (8.3)	276 (10.2)	381 (7.4)		
Other/unknown	99 (1.3)	32 (1.2)	67 (1.3)		
Smoking Status, n (%)				7.25	0.027
Never	840 (10.7)	254 (9.4)	586 (11.3)		
Current	6,267 (79.6)	2,188 (81.0)	4,079 (78.8)		
Past	747 (9.5)	253 (9.4)	494 (9.5)		
Missing	21 (0.3)	7 (0.3)	14 (0.3)		
Pain Intensity, mean (SD)					
Highest Pain Level	6.8 (3.31)	6.7 (3.40)	6.9 (3.25)	3.91	0.048
Closest Pain Level	3.7 (3.68)	3.2 (3.66)	4.0 (3.66)	71.14	< 0.001
Missing	262 (3.3)	99 (3.7)	163 (3.2)		
Pain Diagnoses, n (%)					
Fibromyalgia	241 (3.1)	70 (2.6)	171 (3.3)	3.06	0.080
Headache	733 (9.3)	229 (8.5)	504 (9.7)	3.38	0.066
Limb/extremity, Joint, or Arthritic Pain	3,728 (47.3)	1,045 (38.7)	2,683 (51.9)	123.88	< 0.001
Neuropathic Pain	1,573 (20.0)	430 (15.9)	1,143 (22.1)	42.43	< 0.001
Mental Health Conditions, n (%)					
Major Depressive Disorder	2,014 (25.6)	682 (25.2)	1,332 (25.7)	0.24	0.623
Other Depression	3,787 (48.1)	1,316 (48.7)	2,471 (47.8)	0.62	0.429
Post-Traumatic Stress Disorder	2,068 (26.3)	572 (21.2)	1,496 (28.9)	55.05	< 0.001
Anxiety Disorder	1,421 (18.0)	462 (17.1)	959 (18.5)	2.49	0.115
Bipolar Disorder	1,012 (12.9)	336 (12.4)	676 (13.1)	0.63	0.426
Schizophrenia	793 (10.1)	231 (8.5)	562 (10.9)	10.50	0.001
Substance Use Disorders, n (%)					
Substance Use Disorder (Including Opioid)	7,824 (99.4)	2,672 (98.9)	5,152 (99.6)	13.68	< 0.001
Non-Opioid Substance Use Disorder	4,565 (58.0)	1,611 (59.6)	2,954 (57.1)	4.62	0.032
Alcohol Use Disorder	3,734 (47.4)	1,249 (46.2)	2,485 (48.0)	2.34	0.126
Cocaine	2,578 (32.7)	1,007 (37.3)	1,571 (30.4)	38.37	< 0.001
Cannabis	1,217 (15.5)	409 (15.1)	808 (15.6)	0.32	0.574
Amphetamines	291 (3.7)	145 (5.4)	146 (2.8)	32.28	< 0.001
Sedatives	184 (2.3)	59 (2.2)	125 (2.4)	0.42	0.516
Hallucinogens	33 (0.4)	12 (0.4)	21 (0.4)	0.06	0.803
Receiving Antiretroviral Therapy, n (%)					
Before OUD Diagnosis	---	1,901 (70.4)	---		
After OUD Diagnosis	---	2,108 (78.0)	---		

### Latent Class Solution

The latent class solution with four classes was selected given the lower BIC (6407) than the two (7693) and three (6646) class solutions (Table 2). While the four-class BIC was higher than those of the five (6305) and six (6332) class solutions and the both five- and six-class solutions also met class size criteria of at least 5% prevalence in each class (14.2% and 5.0%, respectively), both the five and six class solutions had one class with very low average posterior probability (60.0% and 59.0%, respectively). The average of the maximum posterior probabilities for the four class solution ranged from 72.0 to 81.0%, with an overall average of 76.0%.

**Table 2:** Fit indices for two through six class models of co-occurring pain, mental health, and substance use diagnoses among patients with OUD

# of classes	Log-Likelihood	AIC	BIC	CAIC	Adjusted BIC	Entropy
2	-50732.77	7462.62	7692.68	7725.68	7587.81	0.65
3	-50133.20	6297.49	6646.06	6696.06	6487.17	0.60
**4**	**-49937.52**	**5940.12**	**6407.21**	**6474.21**	**6194.29**	**0.59**
5	-49810.22	5719.52	6305.12	6389.12	6038.18	0.56
6	-49747.24	5627.56	6331.67	6432.67	6010.72	0.54

AIC, Akaike information criterion; BIC, Bayesian information criterion; CAIC, Consistent Akaike information criterion

### Patterns of Co-occurring Diagnoses across Latent Classes

Based on the prevalence of co-occurring diagnoses in each class, the four identified classes were coined the “Few Co-occurring Diagnoses” (*n* = 3,328; 42.3%); “Multiple Pain Conditions” (*n* = 1,681; 21.3%); “Pain + SUD” (*n* = 1,449; 18.4%); and “Pain + SUD + Mental Health” (*n* = 1,417, 18.0%) (Table 3).

**Table 3:** Demographic and clinical differences between four classes of patients with OUD

Baseline Characteristics	Class 1: Few Co-occurring Diagnoses (*n* = 3,328)	Class 2: Multiple Pain Conditions (*n* = 1,681)	Class 3: Co-occurring Pain + SUD (*n* = 1,449)	Class 4: Co-occurring Pain, SUD, and Mental Health (*n* = 1,417)	Test Statistic	*p*-value
Proportion of Total Sample	42.3%	21.3%	18.4%	18.0%		
Age, mean years (SD)	55.2 (7.76)	55.1 (8.50)	54.8 (6.32)	52.9 (7.99)	31.26	< 0.001
Male Sex, n (%)	3,278 (98.5)	1,623 (96.5)	1,428 (98.6)	1,375 (97.0)	27.70	< 0.001
Race/ethnicity, n (%)					458.03	< 0.001
White	1,014 (30.5)	794 (47.2)	222 (15.3)	506 (35.7)		
Black	1,914 (57.5)	744 (44.3)	1,132 (78.1)	793 (56.0)		
Hispanic	350 (10.5)	118 (7.0)	91 (6.3)	98 (6.9)		
Other/unknown	50 (1.5)	25 (1.5)	4 (0.3)	20 (1.4)		
Smoking Status, n (%)					152.67	< 0.001
Never	309 (9.3)	263 (15.6)	117 (8.1)	151 (10.7)		
Current	2,683 (80.6)	1,174 (69.8)	1,250 (86.3)	1,160 (81.9)		
Past	322 (9.7)	239 (14.2)	82 (5.7)	104 (7.3)		
Missing	14 (0.4)	5 (0.3)	0 (0.0)	2 (0.1)		
Closest Pain Intensity Rating: Moderate to Severe, n (%)	1,376 (41.3)	1,124 (66.9)	587 (40.5)	672 (47.4)	289.30	< 0.001
Missing	216 (6.5)	13 (0.7)	24 (1.7)	9 (0.6)		
Pain Diagnoses, n (%)						
Fibromyalgia	6 (0.2)	168 (10.0)	36 (2.5)	31 (2.2)	370.72	< 0.001
Headache	98 (2.9)	332 (19.8)	88 (6.1)	215 (15.2)	452.47	< 0.001
Limb/extremity, Joint, or Arthritic Pain	863 (25.9)	1,320 (78.5)	659 (45.5)	886 (62.5)	1,400	< 0.001
Neuropathic Pain	65 (2.0)	1,003 (59.7)	146 (10.1)	359 (25.3)	2,400	< 0.001
Mental Health Conditions, n (%)						
Major Depressive Disorder	312 (9.4)	560 (33.3)	219 (15.1)	923 (65.1)	1,800	< 0.001
Other Depression	914 (27.5)	937 (55.7)	701 (48.4)	1,235 (87.2)	1,500	< 0.001
Post-Traumatic Stress Disorder	412 (12.4)	624 (37.1)	355 (24.5)	677 (47.8)	774.62	< 0.001
Anxiety Disorder	216 (6.5)	539 (32.1)	18 (1.2)	648 (45.7)	1,500	< 0.001
Bipolar Disorder	181 (5.4)	171 (10.2)	205 (14.1)	455 (32.1)	645.51	< 0.001
Schizophrenia	214 (6.4)	64 (3.8)	295 (20.4)	220 (15.5)	337.45	< 0.001
Substance Use Disorders, n (%)						
Alcohol Use Disorder	954 (28.7)	328 (19.5)	1,207 (83.3)	1,245 (87.9)	2,700	< 0.001
Cocaine	179 (5.4)	73 (4.3)	1,394 (96.2)	932 (65.8)	5,100	< 0.001
Cannabis	37 (1.1)	72 (4.3)	530 (36.6)	578 (40.8)	1,900	< 0.001
Amphetamines	37 (1.1)	7 (0.4)	17 (1.2)	230 (16.2)	764.87	< 0.001
Sedatives	6 (0.2)	38 (2.3)	12 (0.8)	128 (9.0)	360.77	< 0.001
Hallucinogens	3 (0.1)	0 (0.0)	10 (0.7)	20 (1.4)	51.69	< 0.001
HIV Seropositive, n (%)	1,234 (37.1)	419 (24.9)	566 (39.1)	483 (34.1)	91.55	< 0.001
Receiving Antiretroviral Therapy, n (%)						
Before OUD Diagnosis	773 (62.6)	323 (77.1)	434 (76.7)	371 (76.8)	64.81	< 0.001
After OUD Diagnosis	925 (75.0)	335 (80.0)	462 (81.6)	386 (79.9)	12.96	0.005

**Table 4:** Predictors of buprenorphine initiation and retention (six-month and 12-month) in a multivariable regression analysis

Predictors	Initiated Buprenorphine (*n* = 7,875)^a^			Retained at 6 Months (*n* = 635)^a^			Retained at 12 Months (*n* = 635)^a^		
	n (%)	aOR (95% CI)	p-value	n (%)	aOR (95% CI)	p-value	n (%)	aOR (95% CI)	p-value
Class Membership									
Class 1: Few Co-occurring Diagnoses	330 (9.9)	ref		141 (42.7)	ref		99 (30.0)	ref	
Class 2: Multiple Pain Conditions	158 (9.4)	0.87 (0.68, 1.12)	0.261	72 (45.6)	1.20 (0.75, 1.93)	0.447	44 (27.8)	0.98 (0.59, 1.62)	0.935
Class 3: Pain + SUD	55 (3.8)	0.41 (0.28, 0.59)	< 0.001	21 (38.2)	0.92 (0.44, 1.92)	0.830	15 (27.3)	0.86 (0.38, 1.96)	0.722
Class 4: Pain + SUD + Mental Health	92 (6.5)	0.55 (0.41, 0.75)	< 0.001	41 (44.6)	1.20 (0.67, 2.18)	0.539	26 (28.3)	0.92 (0.48, 1.76)	0.803
Age		0.97 (0.96, 0.98)	< 0.001		1.00 (0.98, 1.02)	0.779		0.99 (0.98, 1.02)	0.870
Sex									
Female	13 (7.6)	ref		5 (38.5)	ref		1 (7.7)	ref	
Male	622 (8.1)	1.21 (0.68, 2.16)	0.520	270 (43.4)	1.13 (0.35, 3.62)	0.833	183 (29.4)	4.74 (0.60, 37.42)	0.140
Race/ethnicity									
White	266 (10.5)	ref		141 (53.0)	ref		94 (35.3)	ref	
Black	307 (6.7)	0.72 (0.60, 0.86)	< 0.001	109 (35.5)	0.48 (0.34, 0.69)	< 0.001	71 (23.1)	0.53 (0.37, 0.78)	0.001
Hispanic	51 (7.8)	0.77 (0.56, 1.06)	0.114	20 (39.2)	0.57 (0.30, 1.06)	0.074	16 (31.4)	0.78 (0.41, 1.50)	0.456
Other/unknown	11 (11.1)	0.99 (0.52, 1.89)	0.971	5 (45.5)	0.73 (0.21, 2.55)	0.625	3 (27.3)	0.68 (0.17, 2.69)	0.581
Smoking Status									
Never	47 (5.6)	ref		15 (31.9)	ref		11 (23.4)	ref	
Current	533 (8.5)	1.70 (1.24, 2.32)	0.001	229 (43.0)	1.88 (0.97, 3.64)	0.059	153 (28.7)	1.38 (0.67, 2.85)	0.378
Past	54 (7.2)	1.44 (0.96, 2.17)	0.081	31 (57.4)	3.18 (1.37, 7.37)	0.007	20 (37.0)	1.94 (0.79, 4.74)	0.148
HIV									
No	427 (8.3)	ref		190 (44.5)	ref		125 (29.3)	ref	
Yes	208 (7.7)	0.96 (0.75, 1.22)	0.724	85 (40.9)	1.11 (0.69, 1.79)	0.653	59 (28.4)	1.15 (0.69, 1.90)	0.597
HIV # Class Membership									
HIV*(Few Co-occurring Diagnoses)		ref			ref			ref	
HIV*(Multiple Pain Conditions)		1.17 (0.75, 1.83)	0.485		0.58 (0.24, 1.42)	0.234		0.53 (0.19, 1.47)	0.224
HIV*(Pain + SUD)		0.77 (0.41, 1.43)	0.404		0.87 (0.25, 3.11)	0.837		1.26 (0.33, 4.86)	0.735
HIV*(Pain + SUD + Mental Health)		1.10 (0.67, 1.83)	0.699		0.54 (0.20, 1.49)	0.235		0.88 (0.30, 2.62)	0.819

^a^Of the 7,875 patients with an index OUD behavior treatment visit during the study period, 635 (8.1%) initiated buprenorphine in the year following the index visit. Among those who initiated buprenorphine, 275 (43.3%) and 184 (29.0%) were retained at six and 12 months, respectively

A plurality of the OUD sample belonged to the “Few Co-occurring Diagnoses” class (42.3%). This class was characterized by a low prevalence of pain, mental health, and SUD diagnoses. Only a quarter of patients had a diagnosis of limb/extremity, joint, or arthritic pain (25.9%), and neuropathic pain was rare (2.0%). The proportion of class members with mental health diagnoses was the lowest of any group, with comparatively low rates of bipolar disorder (5.4%), schizophrenia (6.4%), anxiety disorder (6.5%), and major depressive disorder (9.4%). While nearly a third of patients had a diagnosis of alcohol use disorder (28.7%), other non-opioid SUDs were less common, including cocaine use disorder (5.4%) and cannabis use disorder (1.1%).

The second largest class (“Multiple Pain Conditions” class; 21.3%) was characterized by a high frequency of pain and pain diagnoses, but a generally low frequency of SUDs and other comorbidities. Over two-thirds of patients (66.9%) reported moderate to severe intensity pain closest to the index OUD encounter, and limb/extremity, joint, or arthritic (78.5%) and neuropathic (59.7%) pain diagnoses were present in a majority of class members. Though rates of mental health diagnoses were higher than in the “Few Co-occurring Diagnoses” class, only “other depression” was present in most patients (55.7%), and no single predominant mental health diagnosis emerged among members in this class.

The third class, coined the “Pain + SUD” class (18.4%), was characterized by higher levels of limb/extremity, joint, or arthritic (45.5%) pain as well as SUDs. Cocaine use disorder was present in almost all class members (96.2%), with most also having alcohol use disorder (83.3%) and over a third having cannabis use disorder (36.6%). The prevalence of mental health disorders was lower relative to the “Pain + SUD + Mental Health” class, with the notable exception of schizophrenia (20.4%), which was the highest for any class.

Finally, the smallest class the “Pain + SUD + Mental Health” class (18.0%), was characterized by a high prevalence of co-occurring conditions in all three areas. Limb/extremity, joint, or arthritic pain (62.5%) was common, and over a quarter of patients had neuropathic pain diagnoses (25.3%). The prevalence of alcohol use disorder (87.9%) was the highest of any class, with significant proportions of patients also having cocaine (65.8%) and/or cannabis (40.8%) use disorders. The less common SUDs, including amphetamine (16.2%), sedative (9.0%), and hallucinogen (1.4%) use disorders, were prevalent in this class. Further, a majority of class members had diagnoses of other depression (87.2%) and major depressive disorder (65.1%). Almost half of class members had anxiety disorder (45.7%) and post-traumatic stress disorder (47.8%).

### Patient Characteristics by Class Membership

Nearly every patient characteristic significantly varied by class membership (Table 3). Patients in the “Pain + SUD + Mental Health” class had the lowest average age (52.9 years). The “Multiple Pain Conditions” class was disproportionately White (47.2%), while Black patients were overrepresented in the “Pain + SUD” class (78.1%). Current smoking was most common in the “Pain + SUD” class (86.3%), while past smoking was most common in the “Multiple Pain Conditions” class (14.3%). Only a quarter of patients in the “Multiple Pain Conditions” class had a HIV diagnosis (24.9%), compared with 34.1 − 39.1% of other classes. Among PWH, antiretroviral use varied across classes before (62.6 − 77.1%, Χ^2^ = 64.81, *p* < 0.001) and after OUD diagnosis (75.0 − 81.6%, Χ^2^ = 12.96, *p* = 0.005), with the lowest antiretroviral use in the “Few Co-occurring Diagnoses” class (62.6% and 75.0%, respectively).

### Overall Treatment Initiation and Retention

Fewer than one in ten (8.1%, *n* = 635) patients with a new OUD clinical encounter initiated buprenorphine in the year following the index visit. The rate of buprenorphine initiation did not vary between PWH (7.7%) and PWoH (8.3%, Χ^2^ = 0.741, *p* = 0.389). Among patients who initiated buprenorphine, 43.3% and 29.0% were retained at six and 12 months, respectively, with no differences by HIV status.

### Multivariable Regression of Class Membership and Buprenorphine Outcomes

In the multivariable regression analysis, patients in the two classes with the highest prevalence of co-occurring disorders, the “Pain + SUD” class and the “Pain + SUD + Mental Health” class, were significantly less likely to initiate buprenorphine within one year of the index OUD encounter. Specifically, compared to members of the “Few Co-occurring Diagnoses” class, those in the “Pain + SUD” and “Pain + SUD + Mental Health” classes had 59% (aOR: 0.41; 95% CI: 0.28, 0.59) and 45% (aOR: 0.55; 95% CI: 0.41, 0.75) lower odds, respectively, of initiating buprenorphine (Table 4). Further, after controlling for pain, SUDs, and mental health conditions, Black patients were significantly less likely (aOR: 0.72; 95% CI: 0.60, 0.86) to initiate buprenorphine, while individuals who were documented as currently smoking had 70% higher odds of starting treatment (aOR: 1.70; 95% CI: 1.24, 2.32). Older patients were less likely, albeit by only 3%, to initiate buprenorphine than younger patients (aOR: 0.97; 95% CI: 0.96, 0.98). The association of class membership with buprenorphine initiation did not vary by HIV status.

Among study participants who initiated buprenorphine, class membership and age were not associated with retention in buprenorphine treatment at six or 12 months. Racial differences in retention were evident in multivariable analysis, with Black patients being approximately half as likely to remain in treatment at six months (aOR: 0.48, 95% CI: 0.34, 0.69) and 12 months (aOR: 0.53, 95% CI: 0.37, 0.78). Those reporting past smoking had significantly higher odds of six month (aOR: 3.18; 95% CI: 1.37, 7.37) but not 12 month retention (aOR: 1.94, 95% CI: 0.79, 4.74). As with buprenorphine initiation, neither HIV status nor the interaction of HIV status and class membership were significantly associated with treatment retention (Table 4).

## Discussion

This study used LCA to identify four subgroups of patients with OUD based on patterns of co-occurring pain, non-opioid SUDs, and mental health diagnoses. While the largest class was characterized by low prevalence of diagnoses in all three areas (i.e., “Few Co-occurring Diagnoses” class), other identified classes had higher frequencies of co-occurring diagnoses, including pain alone (i.e., “Multiple Pain Conditions” class) or pain in combination with non-opioid SUDs (i.e., “Pain + SUD” class) with or without mental health conditions (i.e., “Pain + SUD + Mental Health” class). PWH were less likely to present with pain alone and either presented with no conditions or multiple conditions (Table 3).

In multivariable models, patients in the “Pain + SUD” and “Pain + SUD + Mental Health” classes, were less likely to initiate buprenorphine than patients in the “Few Co-occurring Diagnoses” group. There was no difference in buprenorphine initiation between patients in the “Multiple Pain Conditions” and “Few Co-occurring Diagnoses” class. Patients in the “Pain + SUD + Mental Health” class were characterized by a high prevalence of major depressive disorder, anxiety disorder, and/or post-traumatic stress disorder whereas patients in the “Pain + SUD” class had a much lower incidence of mental health conditions. These findings are consistent with several other studies reporting that patients with a mental health condition are less likely to initiate opioid agonist treatment [7, 9, 11, 13]. However, not all studies have come to the same conclusions [8, 10, 15]. These disparate findings may be due to differencs in study designs. Interestingly, the odds of initiating buprenorphine was numerically larger in the “Pain + SUD + Mental Health” class as compared to the “Pain + SUD” class when compared to the “Few Co-occuring Diagnoses” class. This could suggest that a mental health diagnosis in some regard may positively influence treatment initiation. To explore this outcome, we changed the reference class in the regression model to the “Pain + SUD” class. In this model both the “Few Co-occurring Diagnoses” class and “Multiple Pain Conditions” class remained more likely to initiate treatment than the “Pain + SUD” class. However, the difference in the odds of buprenorphine initiation within the “Pain + SUD + Mental Health” class and the “Pain + SUD” class was not statistically significant (aOR: 1.35; 95% CI: 0.88, 2.07).

Overall, the findings from this study suggest that SUD and/or mental health diagnoses may have a negative impact on buprenorphine initiation in the setting of co-occurring pain diagnoses. Others have noted that people with OUD and co-occurring mental health conditions require comprehensive care and lack of this may create access barriers to OUD treatment [48]. Additionally, a lack of mental health support is a commonly cited barrier to buprenorphine prescribing among buprenorphine-waivered physicians [49, 50]. A systems-level approach to coordinating care and expanding mental health resources for patients with co-occurring OUD, mental health conditions, pain, and/or non-opioid SUD diagnoses may improve prescribers’ ability to initiate buprenorphine in these complex patients. Additional research on the impact of expanding buprenorphine access through telehealth during the COVID-19 pandemic may inform future efforts to reduce barriers to buprenorphine initiation among patients with many co-occurring diagnoses.

Retention in buprenorphine treatment at six and 12 months did not vary by class membership, HIV status, or their interaction in the multivariable analysis. This finding adds to a growing body of literature reporting a lack of association between mental health [14, 16, 51] or pain diagnoses [16, 38] and buprenorphine retention. In contrast to previous research which has consistently reported an association between co-occurring SUDs and lower odds of OUD treatment retention, the present study did not find an association between membership in the classes of high SUD prevalence and retention [15, 38, 51–53]. Notably, other studies have rarely controlled for mental health and/or pain diagnoses, which are relevant confounders given that patients with both OUD and pain have higher rates of co-occurring SUD and mental health conditions [54]. The lack of association between class membership and buprenorphine retention suggests that other buprenorphine-related and contextual factors not included in this analysis, including setting of initiation [55], initial and ongoing buprenorphine dosing [55], buprenorphine out-of-pocket costs [56], and provider-level requirements for ongoing care [57], may contribute more substantially to buprenorphine retention than a patient’s overall physical and mental health. Clinical efforts to optimize dosing and clinic requirements during ongoing treatments, combined with policy-level efforts to reduce barriers, including cost, may improve buprenorphine retention among patients with diverse co-occurring diagnoses.

We also found that Black patients were significantly less likely to initiate and continue buprenorphine treatment than their White counterparts, consistent with treatment disparities reported in Medicaid [51, 58], VHA [7, 8, 16, 59], and primary care populations [60–62]. Our study did not include factors associated with buprenorphine retention, such as distance to treatment, housing, transportation or provider, peer, and social support [51], which may have been important covariates, and which could explain this association. Future research is needed to better understand patient-, provider-, and system-level factors that may contribute to the observed racial disparities in buprenorphine treatment engagement, especially given the disproportionate impact of the COVID-19 pandemic among Black patients [63]. Older patients were also less likely to initiate buprenorphine. This disparity has been previously observed in the literature and may be due to unique needs of older adults such as mobility, access to health services, and stigma [64, 65].

This study had several limitations. First, this study focused on opioid agonist therapy, specifically buprenorphine, and did not include methadone, a common methodological decision given that buprenorphine can be prescribed in office-based settings [51–53, 58, 60, 62]. This approach precluded an analysis of overall opioid agonist therapy engagement or the identification of patients who switched from buprenorphine to methadone, although by 2017 the majority of Veterans treated with a medication for OUD were utilizing buprenorphine [36, 37]. Future research is needed to understand whether patterns of comorbidity differentially impact receipt of, and retention on, buprenorphine and methadone. Second, consistent with past research in this study population [8, 14, 40], SUDs were identified using ICD codes, which have high specificity, but low sensitivity compared to self-reported drug use [66]. The prevalence of co-occurring SUDs in this study may therefore be underestimated. However, the inclusion of SUD comorbidities considerably strengthened measures of LCA model adequacy. Third, mental health medications or behavioral treatments were not included as covariates. While this limitation is common in buprenorphine retention literature [8, 16, 53, 61], and the effect of mental health treatment was not found to be significant in a prior study [51], adequate treatment of co-occurring conditions may impact patient engagement with their OUD treatment. Fourth, the study population consisted primarily of older Black men who received OUD behavioral treatment within the VHA population, which may limit the generalizability. Finally, this study did not include contextual variables, including location of residence, homelessness, or social support, nor unmeasured confounders such as OUD severity, that may impact treatment initiation and retention.

## Conclusion

Our results demonstrate heterogeneity in the comorbidity burden of Veterans with OUD. Patients in the two subgroups with the highest comorbidity burden, the “Pain + SUD” and “Pain + SUD + Mental Health” classes, were significantly less likely to initiate buprenorphine than those in the “Few Co-occurring Diagnoses” class. However, among patients who started buprenorphine treatment, patterns of comorbidities were not associated with treatment retention. Other elements, such as systemic and structural factors around race and/or discrimination, may have a greater effect on treatment retention. Future studies are needed to build further understanding of the potential effects of combined co-occurring conditions on OUD-related outcomes.

## Data Availability

The data utilized in this study were derived from the Veterans Aging Cohort Study (VACS) which maintains electronic health record (EHR) from the Veterans Health Administration (VHA). Data is available upon request and proposal approval through the VA Family of EHR Cohorts (VACo Family) website.
